# miR-27a protects human mitral valve interstitial cell from TNF-α-induced inflammatory injury via up-regulation of NELL-1

**DOI:** 10.1590/1414-431X20186997

**Published:** 2018-04-19

**Authors:** Honglei Chen, Zhixu Zhang, Li Zhang, Junzhi Wang, Minghui Zhang, Bin Zhu

**Affiliations:** 1 Department of Cardiology, Chengyang People's Hospital, Qingdao, Shandong, China; 2 Teaching and Research Office of Immunology, Qingdao University, Qingdao, Shandong, China; 3 Department of Imaging, Eastern District of Linyi People's Hospital, Linyi, Shandong, China

**Keywords:** miR-27a, Heart valve disease (HVD), Human mitral valve interstitial cell (hMVIC), TNF-α, Inflammatory injury, NELL-1

## Abstract

MicroRNAs (miRNAs) have been reported to be associated with heart valve disease, which can be caused by inflammation. This study aimed to investigate the functional impacts of miR-27a on TNF-α-induced inflammatory injury in human mitral valve interstitial cells (hMVICs). hMVICs were subjected to 40 ng/mL TNF-α for 48 h, before which the expressions of miR-27a and NELL-1 in hMVICs were altered by stable transfection. Trypan blue staining, BrdU incorporation assay, flow cytometry detection, ELISA, and western blot assay were performed to detect cell proliferation, apoptosis, and the release of proinflammatory cytokines. We found that miR-27a was lowly expressed in response to TNF-α exposure in hMVICs. Overexpression of miR-27a rescued hMVICs from TNF-α-induced inflammatory injury, as cell viability and BrdU incorporation were increased, apoptotic cell rate was decreased, Bcl-2 was up-regulated, Bax and cleaved caspase-3/9 were down-regulated, and the release of IL-1β, IL-6, and MMP-9 were reduced. NELL-1 was positively regulated by miR-27a, and NELL-1 up-regulation exhibited protective functions during TNF-α-induced cell damage. Furthermore, miR-27a blocked JNK and Wnt/β-catenin signaling pathways, and the blockage was abolished when NELL-1 was silenced. This study demonstrated that miR-27a overexpression protected hMVICs from TNF-α-induced cell damage, which might be via up-regulation of NELL-1 and thus modulation of JNK and Wnt/β-catenin signaling pathways.

## Introduction

The human heart is divided into four chambers: two upper atria and two lower ventricles (1). Heart valves, including the mitral valve, tricuspid valve, aortic valve, and pulmonary valve, are present between the atria and ventricle as well as between the ventricle and aorta, allowing blood to flow in only one direction through the heart. Heart valve disease (HVD) is any disease process involving one or more of the four valves of the heart. Acquired HVD occurs largely because of aging, but may also be the result of rheumatic fever, acquired immune deficiency syndrome, endocardial proliferative disorder, antiphospholipid syndrome, ischemic cardiomyopathy, and trauma (2). In patients with acquired HVD, tricuspid valve replacement remains a major surgical intervention. However, a considerable reported risk of mortality (7–22%), and a high incidence of prosthesis-related complications (14–34%) still worry cardiologists, resulting in a high number of reoperations (10–22%) during a follow-up of 5–9 years (3,4). This phenomenon calls for the intense efforts in search of novel effectively therapeutic strategies.

Tumor necrosis factor (TNF) is a proinflammatory cytokine that plays a significant role in a large array of inflammatory diseases such as arthritis and psoriasis (5). TNF overexpression causes a strong inflammation of the aortic and mitral valves, and anti-TNF can be successful in controlling HVD (6). Although the underlying mechanisms of the regulation of TNF expression are not fully understood, a high level of TNF has been identified as a cause of HVD (7).

MicroRNAs (miRNAs) are a class of small, non-coding RNAs with approximately 22 nucleotides that function as post-transcriptional repressors of gene expression, either through degradation of mRNA or translational repression (8). miRNA signatures have been associated with a wide panel of human diseases (9). In the cardiovascular field, circulating miRNAs are currently investigated as biomarkers in acute myocardial infarction and heart failure (10). In addition, an *in vitro* investigation reported that increasing miR-148a-3p levels in human aortic valve interstitial cells was sufficient to repress NF-κB signaling and NF-κB-targeted gene expression (11). Another study indicated that miR-30b was a key regulator of human aortic valvular calcification and apoptosis (12). Although several miRNAs have been reported to be associated with cardiac disease, miRNAs and miRNA regulation are often overlooked as therapeutic targets in HVD (13).

Among the miRNAs, miR-27a has been widely studied due to its critical role in regulating inflammation and innate immune pathway (14–17). Thus, herein, we focused on miR-27a to investigate whether miR-27a also acted as a regulatory molecule that prevented TNF-α induced inflammatory responses in human mitral valve interstitial cells (hMVICs). The findings of this study will provide us with a new perspective that targeting miR-27a may be a potential therapeutic intervention for HVD.

## Material and Methods

### Cell culture and treatment

Normal valves were collected from 5 healthy donor hearts following the donors' deaths in traffic accidents. Of these 5 donors, 3 were males and 2 were females, aged from 33 to 49 years (median, 41 years). This study was approved by the Ethics Committees of the Chengyang People's Hospital. Written consent was obtained from all donors' parents or spouse. hMVICs were isolated by collagenase digestion and cultured in Dulbecco's modified eagle medium (DMEM; Hyclone, USA) supplemented with 10% fetal bovine serum (FBS; Hyclone) and 4 mmol/L L-glutamine as previously described (18). Cells were maintained in an incubator at 37°C and 5% CO_2_.

TNF-α (Sigma-Aldrich, USA) at a concentration of 40 ng/mL was used for treating cells for 48 h.

### Cell transfection

miR-27a mimic, miR-27a inhibitor, and their negative control (NC, scrambled) were synthesized by GenePharma Co. (China). For overexpressing NELL-1, full-length wild type NELL-1 sequences were constructed into pcDNA3.1 plasmids (GenePharma). Empty pcDNA3.1 plasmid was used as a blank control. For NELL-1 silencing, NELL-1-targeted specific siRNAs (GenePharma) were transfected into cells. Non-targeting sequences were used as a blank control. Cell transfections were conducted using Lipofectamine 3000 reagent (Invitrogen, USA) following the manufacturer's protocol. At 48 h of transfection, 0.5 mg/mL G418 (Sigma-Aldrich) was added to the culture medium for selection of stably transfected cells. After approximately 4 weeks, G418-resistant cell clones were established and were collected for use in the following experiments.

### Cell viability assay

Transfected cells were planted on 24-well plates at a density of 1×10^5^ cells per well. After treatment with TNF-α for 48 h, cells were collected and stained with 1 mL trypan blue solution (Beyotime, China). The cell resuspension solution was allowed to culture at room temperature for 5 min, and then stained cells were counted under a hemocytometer. Viability was calculated as % cell viability = living/total cells ×100%.

### Cell proliferation

Cell proliferation was assessed by bromodeoxyuridine (BrdU) incorporation assay. Transfected cells were planted in 96-well plates at a density of 1×10^3^ cells per well. After adherence, 20 mL BrdU (Abcam, USA) with dilution of 1:500 was added to each well, followed by a 24-h incubation at 37°C. Then, anti-BrdU antibody, peroxidase-conjugate goat anti-mouse IgG antibody, TMB peroxidase substrate, and stop solution were added. Immune complexes were measured by photometric detection at 690 nm.

### Apoptosis assay

Quantitation of apoptotic cells were performed using Annexin V-FITC/PI apoptosis detection kit (Beijing Biosea Biotechnology, China). In brief, the transfected cells were planted in 6-well plates at a density of 1×10^6^ cells per well and were subjected to TNF-α for 48 h. Cells were then collected and resuspended in 200 μL binding buffer (Beijing Biosea Biotechnology, China) containing 10 μL Annexin V-FITC. After 30 min incubation in the dark at room temperature, 5 μL PI and 300 μL binding buffer were added, and the samples were immediately analyzed on a flow cytometer (BD Biosciences, USA). Annexin-V positive cells were recognized as apoptotic cells.

### Enzyme-linked immunosorbent assay (ELISA)

After transfection and TNF-α exposure, cell culture supernatant was collected and concentrations of inflammatory cytokines were measured by ELISA kits (R&D Systems, Abingdon, UK) according to the manufacturer's protocols.

### qRT-PCR

Total RNAs of cells were extracted by TRIzol (Invitrogen, USA) according to the manufacturer's instructions. miRNAs were extracted using miRNeasy Mini Kit (Qiagen, China). cDNA was synthesized from 1 μg of total RNAs using the PrimeScript Reverse Transcriptase (Takara, China) and random hexamers or oligo (dT). qRT-PCR was performed on QuantStudio 6 Flex Realtime PCR system (Applied Biosystems, USA) with the specific primer for hsa-miR-27a (forward: 5′-GGG TTC ACA GTG GCT AAG-3′ and reverse: 5′-CAG TGC GTG TCG TGG AGT-3′) and NELL-1 (forward: 5′-TAT GAG CGT GTG ATA GAC CCT C-3′ and reverse: 5′-TCC CAT CTT GGA TGA TCC CTT-3′). GAPDH (forward: 5′- GTC TCC TCT GAC TTC AAC AGC G-3′ and reverse: 5′- ACC ACC CTG TTG CTG TAG CC-3′) and U6 (forward: 5′-CTC GCT TCG GCA GCA CA-3′ and reverse: 5′-AAC GCT TCA CGA ATT TGC GT-3′) were used as the internal reference for NELL-1 and miR-27a, respectively. Data were calculated by the 2^-ΔΔCt^ method (19).

### Western blot

Total cellular protein used for western blotting was extracted using 1% Triton X-100 and 1 mM PMSF, pH 7.4, over ice for 30 min. The protein concentration was determined by the BCA™ Protein Assay Kit (Pierce, USA). Protein (0.1 mg) was resolved over 10–12% SDS-PAGE and transferred to a polyvinylidene fluoride (PVDF) membrane. Following blockage in 5% non-fat dry milk/0.05% Tween 20 (in 20 mM TBS, pH 7.6) for 1 h at room temperature, the PVDF membranes were probed by primary antibodies overnight at 4°C. Anti-Bcl-2 (1:1000 dilution, ab59348), anti-Bax (1:1000 dilution, ab53154), anti-caspase-3 (1:250 dilution, ab13586), anti-caspase-9 (1:200 dilution, ab25758), anti-IL-1β (1:250 dilution, ab200478), anti-IL-6 (1:1000 dilution, ab208113), anti-MMP-9 (1:1000 dilution, ab38898), anti-Wnt3a (1:1000 dilution, ab169175), anti-Wnt5a (1:1000 dilution, ab179824), anti-β-catenin (1:4000 dilution, ab6302), anti-NELL-1 (1:1000 dilution, ab197315), and anti-GAPDH (1:1000 dilution, ab8245) were purchased from Abcam (USA). Anti-JNK (1:500 dilution, sc-7345), anti-p-JNK (1:1000 dilution, sc-293136), anti-c-JUN (1:200 dilution, sc-166540), and anti-p-c-JUN (1:200 dilution, sc-53182) were obtained from Santa Cruz Biotechnology (USA). Then, the PVDF membranes were incubated with goat anti-rabbit (1:5000 dilution, ab6720, Abcam) and goat anti-mouse (1:5000 dilution, ab6788, Abcam) secondary antibodies for 1 h at room temperature. Blots were visualized by enhanced chemiluminescence (ECL) method. Protein bands were quantified by the ImageJ 1.49 (National Institutes of Health, USA).

### Statistical analysis

All experiments were repeated three times. The results of multiple experiments are reported as means±SD. Statistical analyses were performed using SPSS 19.0 statistical software (SPSS Inc., USA). P-values were calculated using one-way analysis of variance (ANOVA) or Student's *t*-test, followed by the Kolmogorov-Smirnov and Shapiro-Wilk tests for the detection of normality of data. A P-value of <0.05 was considered to be statistically significant.

## Results

### TNF-α induced inflammatory injury in hMVICs

**Figure 1.**
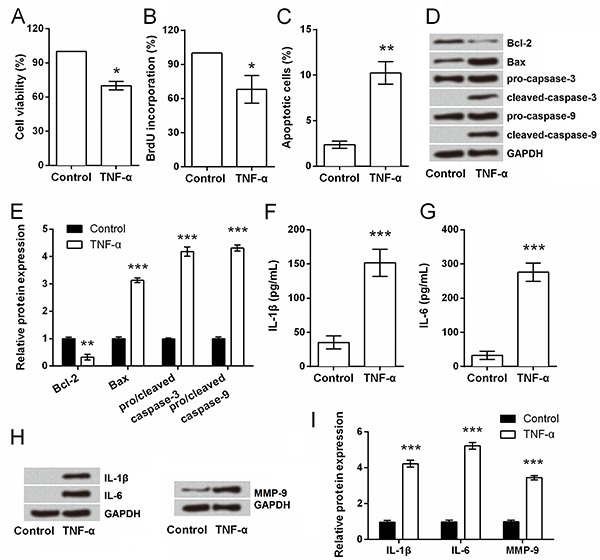
TNF-α impaired cell proliferation, induced apoptosis, and increased the release of proinflammatory cytokines in human mitral valve interstitial cells (hMVICs). *A*, Cell viability; *B*, BrdU incorporation; *C*, apoptotic cell rate; *D* and *E*, expressions of apoptosis-related proteins; *F*, IL-1β and *G*, IL-6 secretions, and *H* and *I*, protein expressions of proinflammatory cytokines were assessed after hMVICs were subjected to 40 ng/mL TNF-α for 48 h. Data are reported as means±SD (n=3). *P<0.05, **P<0.01, ***P<0.001 *vs* control group (Student's *t*-test).

hMVICs were subjected to 40 ng/mL TNF-α for 48 h, and then the changes in cell proliferation, apoptosis, and inflammatory cytokine expressions were monitored. As shown in Figure 1A-C, cell viability and BrdU incorporation were significantly reduced (P<0.05), and apoptotic cell rate (P<0.01) was significantly increased in response to TNF-α stimulation. Down-regulation of Bcl-2 (an anti-apoptotic protein), up-regulation of Bax (a pro-apoptotic protein), and activations of caspase-3 and -9 were found in TNF-α treated cells (P<0.01 or P<0.001, Figure 1D and E). Concentrations of proinflammatory cytokines, IL-1β, and IL-6 in the culture supernatant were much higher in the TNF-α group compared to the control group (P<0.001, Figure 1F and G). Protein expressions of IL-1β, IL-6, and MMP-9 were highly expressed in TNF-α treated cells (P<0.001, Figure 1H and I). These data suggested that TNF-α induced significant inflammatory injury in hMVICs.

### TNF-α down-regulated miR-27a expression in hMVICs

**Figure 2.**
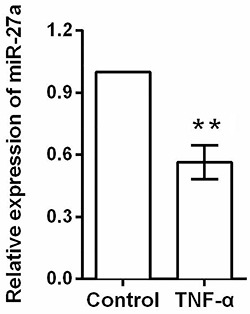
TNF-α down-regulated miR-27a expression in human mitral valve interstitial cells. The expression level of miR-27a in cells was measured after TNF-α exposure. Data are reported as means±SD (n=3). **P<0.01 *vs* control group (Student's *t-*test).

We evaluated the expression change of miR-27a after TNF-α exposure and found that miR-27 expression level was significantly reduced in the TNF-α group compared to the control group (P<0.01, Figure 2). Thus, we inferred that miR-27a might be implicated in TNF-α-induced inflammatory injury in hMVICs.

### miR-27a protected hMVICs from TNF-α-induced inflammatory injury

**Figure 3.**
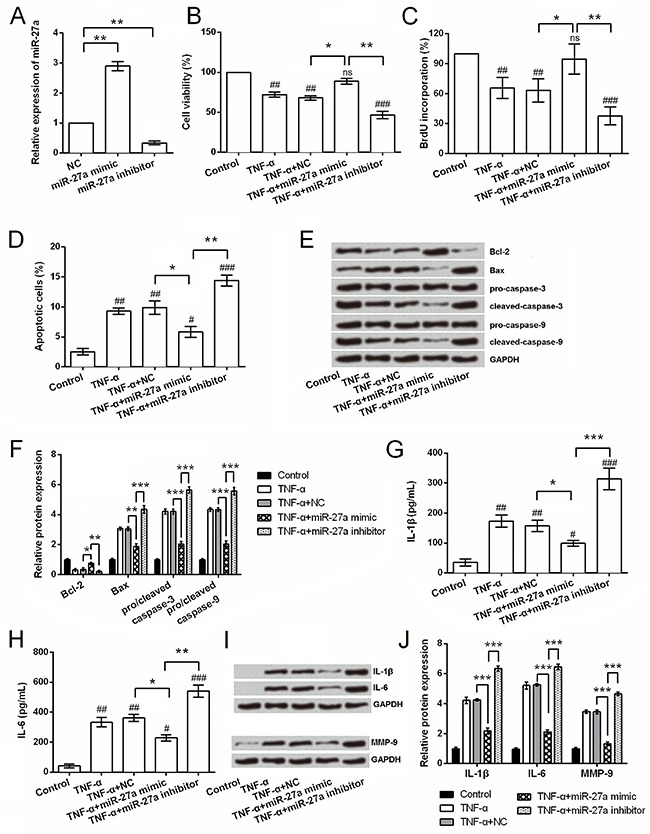
miR-27a up-regulation recovered the impairment of cell proliferation, induced apoptosis, and released proinflammatory cytokines induced by TNF-α in human mitral valve interstitial cells (hMVICs). *A*, The expression of miR-27a was detected after hMVICs were transfected with miR-27a mimic, miR-27a inhibitor, or the negative control (NC); *B*, cell viability; *C*, BrdU incorporation; *D*, apoptotic cell rate; *E* and *F*, expressions of apoptosis-related proteins; *G*, IL-1β and *H*, IL-6 secretions, and *I* and *J*, protein expressions of proinflammatory cytokines were assessed after hMVICs were transfected with miR-27a mimic/inhibitor and exposed to TNF-α. Data are reported as means±SD (n=3). *P<0.05, **P<0.01, ***P<0.001 *vs* the indicated group (ANOVA). ^#^P<0.05, ^# #^P<0.01, ^# ##^P<0.001 *vs* control group (ANOVA). ns: not significant.

To validate the importance of miR-27a in TNF-α-injured hMVICs, miR-27a mimic and miR-27a inhibitor were transfected into hMVICs. Transfection efficiency was detected by qRT-PCR and results showed that cells transfected with the miR-27a mimic exhibited significant overexpression compared with the NC group, while transfection with the miR-27a inhibitor resulted in a significant decrease in expression (both P<0.01, Figure 3A). Then, we found that miR-27a overexpression notably recovered TNF-α-induced inflammatory injury, as cell viability and BrdU incorporation were increased, apoptotic cell rate was reduced, Bcl-2 was up-regulated, and Bax, cleaved capsase-3 and -9 were down-regulated. The levels of IL-1β, IL-6, and MMP-9 were decreased by addition of miR-27a mimic (P*<*0.05, P<0.01 or P*<*0.001, Figure 3B-J). In contrast, miR-27a suppression aggravated TNF-α-induced inflammatory injury, as miR-27a inhibitor affected cell viability, BrdU incorporation, apoptosis, and the release of proinflammatory cytokines resulted in the opposite impacts.

### miR-27a blocked JNK and Wnt/β-catenin signaling pathways

**Figure 4.**
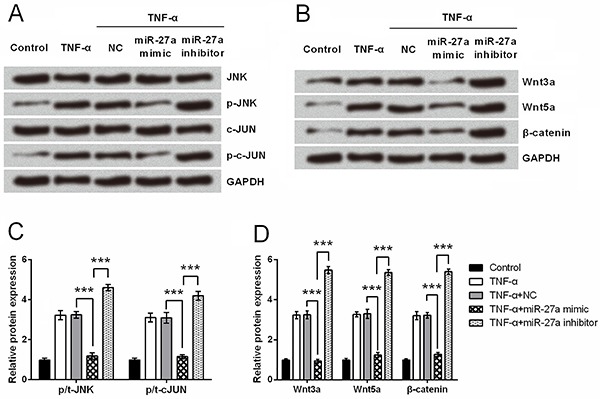
miR-27a blocked JNK and Wnt/β-catenin signaling pathways. *A* and *C*, Expressions of main proteins in JNK signaling, and *B* and *D*, expressions of main proteins in Wnt/β-catenin signaling were measured after human mitral valve interstitial cells were transfected with miR-27a mimic/inhibitor and exposed to TNF-α. Data are reported as means±SD (n=3). ***P<0.001 *vs* the indicated group (ANOVA).

To further reveal the molecular mechanisms by which miR-27a protected hMVICs from TNF-α-induced inflammatory injury, we focused on JNK and Wnt/β-catenin pathways. As shown in Figure 4A-D, JNK and Wnt/β-catenin signaling pathways were activated by TNF-α stimulation, as evidenced by the up-regulations of p-JNK, p-c-JUN, Wnt3a, Wnt5a, and β-catenin (P<0.001). miR-27a overexpression could partially abolish these up-regulations, which were induced by TNF-α; as expected, miR-27a suppression aggravated it.

### NELL-1 was positively regulated by miR-27a

**Figure 5.**
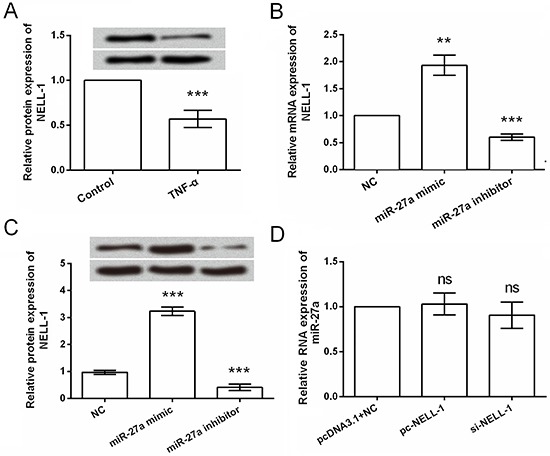
NELL-1 was positively regulated by miR-27a. *A*, Protein expression of NELL-1 in TNF-α-treated cells. *B*, mRNA and *C*, protein expression levels of NELL-1 in hMVICs were determined after human mitral valve interstitial cells (hMVICs) were transfected with miR-27a mimic, miR-27a inhibitor, or the negative control (NC). *D*, RNA level of miR-27a in hMVICs transfected with pc-NELL-1 (a NELL-1-expressing vector) or si-NELL-1 (a siRNA specific for NELL-1). Data are reported as means±SD (n=3). **P<0.01, ***P<0.001 *vs* control, NC, or pc-DNA3.1+NC group (ANOVA). ns: not significant.

A previous study reported that NELL-1 could significantly attenuate BMP2-induced inflammation (20). Here, we assessed whether miR-27a exerted anti-inflammatory properties in a NELL-1-dependent manner. We found that protein expression of NELL-1 was reduced in response to TNF-α stimulation (P*<*0.001, Figure 5A). By performing qRT-PCR and western blot analyses, we found that both the mRNA and protein levels of NELL-1 were up-regulated by transfection with miR-27a mimic, while down-regulated by miR-27a inhibitor transfection (P<0.01 or P<0.001, Figure 5B and C). Interestingly, the RNA level expression of miR-27a was unaffected by NELL-1 alteration (P>0.05, Figure 5D).

### NELL-1 protected hMVICs from TNF-α-induced inflammatory injury

**Figure 6.**
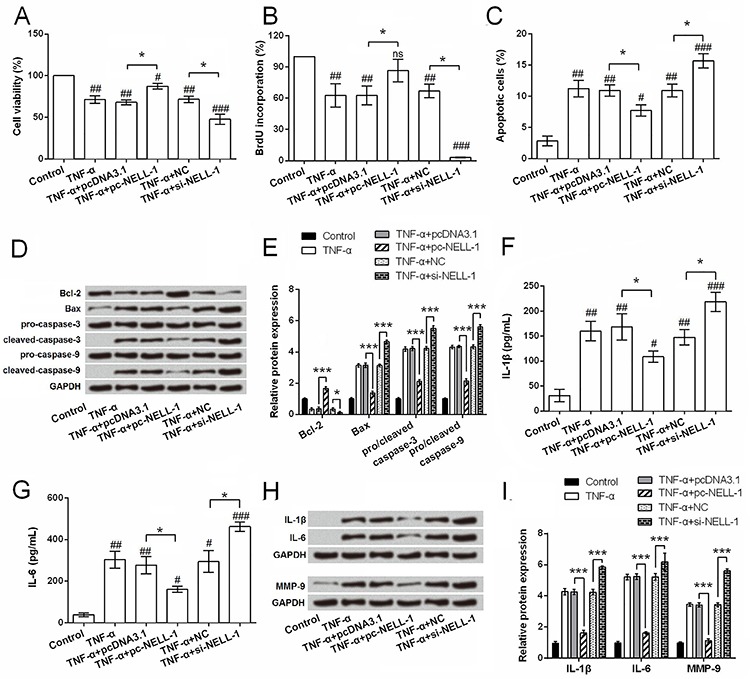
NELL-1 recovered the impairment of cell proliferation, the induction of apoptosis, and the release of proinflammatory cytokines induced by TNF-α in human mitral valve interstitial cells (hMVICs). *A*, Cell viability; *B*, BrdU incorporation; *C*, apoptotic cell rate; *D* and *E*, expressions of apoptosis-related proteins; *F*, IL-1β and *G*, IL-6 secretions, and *H* and *I*, protein expressions of proinflammatory cytokines were assessed after hMVICs were transfected with pc-NELL-1/si-NELL-1 and exposed to TNF-α. Data are reported as means±SD (n=3). *P<0.05, ***P<0.001 *vs* the indicated group (ANOVA). ^#^P<0.05, ^# #^P<0.01, ^# ##^P<0.001 *vs* control group (ANOVA). ns: not significant.

NELL-1 was overexpressed in hMVICs by transfection with pc-NELL-1 (a NELL-1-expressing vector) and was suppressed by transfection with si-NELL-1 (a specific siRNA for NELL-1) (Supplementary Figure S1). The functional effects of NELL-1 on hMVICs were then investigated. As shown in Figure 6A-I, NELL-1 up-regulation exhibited similar effects as miR-27a overexpression as it significantly promoted cell viability and BrdU incorporation, suppressed apoptosis, and reduced the release of IL-1β, IL-6, and MMP-9 (P<0.05 or P<0.001). As expected, NELL-1 silence affected hMVICs resulted in the opposite impacts. We also found that the protective functions of miR-27a in TNF-α-injured hMVICs were abolished when NELL-1 was silenced (P<0.05, Supplementary Figure S2). In contrast, the protective functions of miR-27a were enhanced by NELL-1 overexpression (P<0.05 or P<0.01, Supplementary Figure S3).

### miR-27a blocked JNK and Wnt/β-catenin signaling pathways in a NELL-1-dependent manner

**Figure 7.**
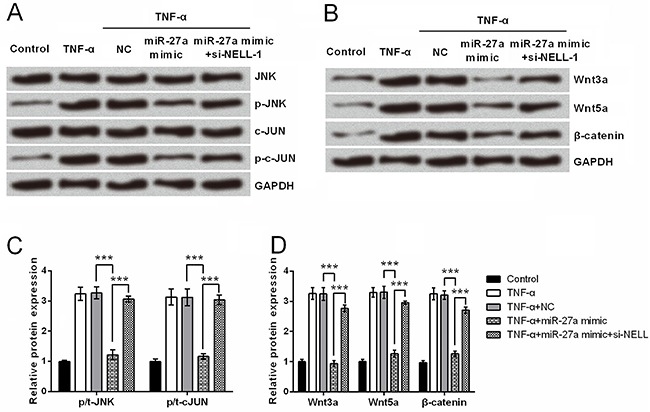
miR-27a blocked JNK and Wnt/β-catenin signaling pathways in a NELL-1-dependent manner. *A* and *C*, Expressions of main proteins in JNK signaling, and (*B* and *D*) expressions of main proteins in Wnt/β-catenin signaling were measured, after human mitral valve interstitial cells were co-transfected with miR-27a mimic and si-NELL-1, and were exposed to TNF-α. Data are reported as means±SD (n=3). ***P<0.001 *vs* the indicated group (ANOVA).

Furthermore, miR-27a mimic and si-NELL-1 were co-transfected into hMVICs, and the expression changes of core proteins in JNK and Wnt/β-catenin pathways were reassessed. As shown in Figure 7A-D, miR-27a overexpression did not attenuate TNF-α-activated JNK and Wnt/β-catenin pathways when NELL-1 was knocked down. This data indicated that miR-27a blocked JNK and Wnt/β-catenin signaling pathways possibly in a NELL-1-dependent manner.

## Discussion

miRNAs are now thought to be key regulators of pathological processes in cardiovascular diseases, including HVD (13). Despite the fact that miR-27a has been reported as a critical regulator in inflammatory responses, no study has focused on the regulatory effects of miR-27a on HVD. Herein, hMVICs were cultured with 40 ng/mL TNF-α, and the effects of miR-27a dysregulation on TNF-α-induced inflammatory injury were evaulated. We found that miR-27a was down regulated after stimulation by TNF-αin hMVICs. miR-27a overexpression rescued hMVICs from TNF-α-induced inflammatory injury, as cell viability and BrdU incorporation were increased, apoptotic cell rate was decreased, Bcl-2 was up-regulated, Bax and cleaved caspase-3/9 were down-regulated, and the release of IL-1β, IL-6, and MMP-9 was reduced when miR-27a was overexpressed. NELL-1 was positively regulated by miR-27a, and NELL-1 up-regulation exerted the similarly protective functions as miR-27a overexpression on TNF-α-induced cell damage. We also found that miR-27a overexpression blocked JNK and Wnt/β-catenin signaling pathways, and the blockage was mediated by NELL-1 expression.

In valvular heart diseases, miRNAs have been proposed as potential hallmarks. Oury et al. (21) mentioned that circulating miR-27a was increased in patients with aortic stenosis. Nigam et al. (22) reported that miR-27a was decreased in human bicuspid aortic tissue obtained from patients who needed aortic valve replacement. These previous studies point out that miR-27a expression may be implicated in valvular heart diseases. Herein, our research demonstrated that miR-27a was down regulated in hMVICs upon TNF-α stimulation, which provided evidence that miR-27a might be a regulatory molecule involved in HVD.

Currently, miR-27a has been widely studied due to its controversial role in inflammatory responses. Some investigations indicated that miR-27a exhibited anti-inflammatory functions. For example, overexpression of miR-27a could decrease the production of inflammatory cytokines, such as IL-6, IL-1β, TNF-α, and nitric oxide (NO) in lipopolysaccharide (LPS)-stimulated microglia (14). miR-27a overexpression has also been reported to attenuate ischemia reperfusion-induced inflammatory damage to the blood-spinal cord barrier by inhibiting the NF-κB/IL-1β pathway (16). Nevertheless, several other reports indicated the proinflammatory functions of miR-27a. In macrophages, miR-27a overexpression enhanced the expression of proinflammatory cytokines (IL-1β, IL-6, IL-12, and TNF-α), and diminished the expression of anti-inflammatory cytokine IL-10 (17). Also in macrophages, mmu-miR-27a was indicated as a proinflammatory gene, as its target gene MCPIP1 could decrease the secretion of IL-6, IL-1β, and IL-10, which were stimulated by LPS (15). These data indicated the complex and critical roles of miR-27a in inflammatory responses. We speculated that the discrepancy may be caused by the different cell types and stimulating conditions that were used. In the current study, our data indicated the anti-inflammatory roles of miR-27a in hMVICs, which were injured by TNF-α. This finding provided the first evidence that miR-27a overexpression acted as a regulatory mechanism that prevented TNF-α-driven inflammatory responses.

NELL-1, a uniquely secreted protein of 810 amino acids, was first studied in the context of human craniofacial skeletal development (23). *In vivo* investigations have demonstrated that NELL-1 has the capacity to drive the growth and differentiation of bone and cartilage tissue (24–26). A previous study has also provided evidence that NELL-1 has anti-inflammatory properties, as it suppressed acute and chronic inflammatory cell infiltration, inflammatory cytokine production, soft tissue exudate, and even systemic levels of inflammatory markers, which were induced by BMP2 (20). In the current study, NELL-1 was positively regulated by miR-27a, and the protective functions of NELL-1 up-regulation in TNF-α-injured hMVICs were observed. Our findings were consistent with a previous study and confirmed the anti-inflammatory role of NELL-1. Based on the data in this study, we also inferred that miR-27a rescued hMVICs from TNF-α-induced inflammatory injury, which might have been via up-regulation of NELL-1.

The JNK and Wnt/β-catenin pathways act as critical intermediates and convergence points in immune system signaling (27,28). These two signaling pathways can also regulate cell proliferation and apoptosis (29–31). It is widely accepted that JNK and Wnt/β-catenin signaling pathways can be activated by TNF-α treatment (32). This was also confirmed in our study, in which Wnt3a, Wnt5a, β-catenin and the phosphorylated forms of JNK and c-JUN were all highly expressed in response to TNF-α exposure in hMVICs. We also found that miR-27a overexpression blocked JNK and Wnt/β-catenin signaling pathways. However, miR-27a overexpression did not block these two pathways when NELL-1 was knocked down. Based on these data, we speculated that miR-27a modulated JNK and Wnt/β-catenin signaling pathways in a NELL-1-dependent manner.

In conclusion, this study demonstrated that miR-27a overexpression protected hMVICs from TNF-α-induced cell damage, which might be via up-regulation of NELL-1 and thereby modulation of JNK and Wnt/β-catenin signaling pathways. This study indicated that therapies designed to up-regulate miR-27a may help HVD patients to effectively alleviate the illness.

## Supplementary material

Click here to view [pdf].
